# Postoperative Respiratory Failure in US Pediatric Care: Evidence from a Nationally Representative Database

**DOI:** 10.3390/pediatric17030058

**Published:** 2025-05-14

**Authors:** Michael Samawi, Gulzar H. Shah, Linda Kimsey

**Affiliations:** Department of Health Policy and Community Health, Jiann-Ping Hsu College of Public Health Georgia Southern University, P.O. Box 8015, Statesboro, GA 30460, USA; msamawi1@georgiasouthern.edu (M.S.); lkimsey@georgiasouthern.edu (L.K.)

**Keywords:** postoperative respiratory failure, quality improvement, pediatric care, pediatric adverse events, kid’s inpatient database, hospital and patient characteristics

## Abstract

**Background/Objectives**: Pediatric postoperative respiratory failure in the United States is increasingly considered a significant adverse event due to the increased risk of co-morbidities, suffering, and cost of healthcare. This study investigates associations between pediatric adverse events (PAEs) and hospital and patient characteristics within the inpatient hospital setting, focusing solely on the framework of pediatric quality indicators (PDIs) from the Agency for Healthcare Research and Quality (AHRQ). Specifically, the study focuses on PDI 09-Postoperative Respiratory Failure (PORF). **Methods**: This quantitative research analyzed the inpatient discharge data from the Healthcare Cost and Utilization Project (HCUP) Kids’ Inpatient Databases (KID) for 2019. We performed multivariate logistic regression to analyze patient-level encounters with PORF. **Results**: The results indicate that smaller, rural, and non-teaching hospitals exhibit significantly lower odds of PDI 09 than large, urban, and urban teaching hospitals, reflecting a concentration of operative procedures. In comparison, the Western United States exhibits higher odds of PDI 09. Various individual factors such as gender, age, race, service lines, payment sources, and major operating room procedures demonstrate differing levels of significance concerning PDI 09, warranting further investigation into confounding factors. In contrast, hospital ownership consistently shows lower odds of PORF risk for private, investor-owned hospitals. **Conclusions**: This study provides contextual expansion on the findings and offers valuable insights into PAEs in the inpatient hospital setting. It highlights areas for developing evidence-based interventions and guidelines for clinicians and policymakers. Ultimately, the findings contribute to the growing understanding of factors influencing PORF and emphasize the importance of targeted strategies for improving pediatric patient safety.

## 1. Introduction

Postoperative respiratory failure (PORF) in pediatric patients is generally defined as the inability to wean from mechanical ventilation within 48 h of surgery or the need for unplanned post-surgical re-intubation [1]. This severe complication reflects a failure of pulmonary recovery and often necessitates intensive care interventions, leading to substantial morbidity, mortality, and resource utilization [1]. Accordingly, the Agency for Healthcare Research and Quality (AHRQ) developed Pediatric Quality Indicator 09 (PDI 09) to monitor PORF, identifying it as a potentially preventable adverse postoperative event [2,3].

Since its introduction in 2006, the PDI system has been refined through risk adjustment protocols, reference populations, and updated ICD-10 coding guidelines [2]. These tools enable robust analysis of hospital- and area-level data to detect patterns of preventable complications, including PORF. Among all PDIs tracked in the 2019 State Inpatient Database (SID), PDI 09 had the highest reported rate—26.48 per 1000 discharges—highlighting its importance as a pediatric safety indicator [2].

Further national estimates suggest an incidence of 267.5 rate per 10,000 pediatric discharges, indicating that PORF affects a clinically meaningful subset of pediatric surgical patients, with some studies showing rates ranging from 1.31% to 1.41% in children under 18 years of age between 2012 and 2014 [3,4]. When PORF occurs, its impact is serious. Recent analyses using national databases reported that pediatric acute respiratory failure hospitalizations are associated with a median hospital stay of 15 days and an average cost of USD 77,168 per admission [5]. These findings underscore the substantial clinical and financial burden associated with PORF in pediatric inpatient care.

A growing body of research has attempted to identify clinical and demographic risk factors for PORF to guide preventive strategies. Risk factors include very young age—particularly neonates and infants—chronic lung disease (e.g., asthma, bronchiolitis), neuromuscular disorders, and exposure to major surgical procedures such as abdominal or spinal operations [5,6]. Disease severity indicators such as high ASA (American Society of Anesthesiologists) physical status and composite comorbidity scores further increase PORF risk [4,7,8]. Mechanical ventilation is both a defining element and a risk factor for PORF, especially in cases of residual neuromuscular blockade or prolonged perioperative support [8].

Efforts to prevent PORF in pediatric populations center on optimal perioperative management, though evidence is still limited. Recommended strategies include careful ventilator control, effective pain management without oversedation, and postoperative pulmonary hygiene measures such as incentive spirometry or chest physiotherapy [8]. However, many such practices are adapted from adult care, and high-quality pediatric-specific clinical trials remain lacking [1,8].

Recent studies have highlighted the relevance of unplanned endotracheal tube intubation (UPI) as a related and predictive marker for PORF risk. For instance, predictors of UPI in pediatric noncardiac surgery included age under one year and prolonged operative time [7]. In a large national sample, other predictors of UPI within 72 h included severe cardiac risk factors, ASA classification, central nervous system tumors, developmental delays, malignancy, and neonate status [8]. Early UPI was associated with increased 30-day mortality, particularly in neonates, due to physiologic vulnerabilities such as diaphragmatic fatigue and increased closing volumes. These findings emphasize the importance of early identification of high-risk patients and the application of preventive interventions like noninvasive ventilation and chest physiotherapy [8].

In a previous study, we identified elevated odds of neonatal bloodstream infections (NBSI/NQI 03) in large, urban teaching hospitals and among specific demographic groups [9]. Building on this work, the current study uses the AHRQ PDI 09 classification to investigate PORF within a nationally representative dataset. The goal is to further map out risk factors and institutional patterns in pediatric adverse events using an updated and high-incidence indicator.

Therefore, to advance understanding of PORF, this study analyzes data from the 2019 HCUP Kids’ Inpatient Database (KID), the largest all-payer pediatric discharge dataset in the United States (~3 million discharges annually) [10]. We apply the AHRQ’s PDI 09 definitions to identify cases and examine associations with hospital- and patient-level characteristics. This leads to the following research questions:

1. Are specific hospital characteristics (region, teaching status, rurality, ownership, size) associated with the likelihood of pediatric PORF?
2. Are specific patient characteristics (age, gender, payer, race/ethnicity, service line of care, or major surgical procedure) associated with PORF?

Answering these questions will help identify high-risk populations and healthcare settings and inform targeted quality improvement strategies for pediatric surgical care.

## 2. Materials and Methods

This study is an observational, population-based retrospective cohort analysis utilizing data from the 2019 HCUP Kids’ Inpatient Database (KID), provided by the Agency for Healthcare Research and Quality (AHRQ) [10]. The HCUP-KID is the most comprehensive all-payer pediatric inpatient care database in the United States, capturing approximately three million pediatric discharges annually from 48 states and the District of Columbia. It includes discharge-level and hospital-level data from community, non-federal, short-term general and specialty hospitals (excluding rehabilitation and long-term care facilities). Data from the 2019 release were used, which incorporates ICD-10-CM/PCS coding following the 2015 transition.

This retrospective design enables national-level estimation of pediatric hospital stays and allows exploration of the incidence and contributing factors associated with PORF. The study uses AHRQ’s Pediatric Quality Indicators (PQIs), particularly PDI 09, to identify relevant adverse events using SAS QI^®^ v2020 software (SAS 9.4) and ICD-10 code-based definitions (see Appendix A for full ICD-10 code criteria for PDI 09).

### 2.1. PICO Framework

- Population: Pediatric inpatients aged 0–20 years included in the 2019 HCUP KID database.
- Intervention/Exposure: Patient and hospital characteristics (e.g., region, teaching status, payer, service line, prior major surgery).
- Comparison: Groups stratified by hospital features (e.g., public vs. private, rural vs. urban) and patient factors (e.g., male vs. female, different service lines).
- Outcome: Occurrence of postoperative respiratory failure, defined by AHRQ’s PDI 09 measure.

### 2.2. Variables

PORF cases included in this study are identified using the AHRQ-defined PDI 09 measure, which captures potentially preventable adverse events following surgery. The classification does not distinguish between expected postoperative complications, unforeseen physiological responses, or errors in care. However, PDI 09 was designed to signal outcomes that may be influenced by modifiable care practices [2]. As such, while PORF events are not inherently indicative of malpractice, they represent important markers for safety and quality monitoring. In this analysis, PORF events are examined in relation to both hospital and patient characteristics to identify institutional patterns and at-risk populations.

These hospital characteristics are used as proxies for institutional resources and care complexity. For example, teaching hospitals may care for more medically complex patients, while regional and ownership differences may reflect variability in access to subspecialty care or resource allocation. However, the KID dataset does not include hospital-level data on ICU availability, ventilator types, or clinical protocols such as early warning systems (MEWS/EWS), which may also affect outcomes [10].

The primary outcome variable is the presence of PDI 09, which captures postoperative respiratory failure using AHRQ specifications. It is coded as a binary variable (1 = Yes, 0 = No). Independent variables are grouped into two domains:

- Hospital characteristics: region (Northeast, Midwest, South, West), location and teaching status (rural, urban non-teaching, urban teaching), bed size (small, medium, large), and ownership (public, private non-profit, private for-profit).
- Patient demographics: age, sex (male = 0, female = 1), race/ethnicity (White, Black, Hispanic, Asian/Pacific Islander, Native American, Other), payer type (government, private, self-pay), and surgical procedure (Yes/No).

Additionally, discharges are grouped by clinical service line: maternal/neonatal, mental health/substance use, injury, surgical, or medical. While KID includes indicators for surgical procedures and DRG classifications, variables such as elective versus emergency procedures, prior hospitalization history, hereditary or immunologic conditions, and detailed metabolic or neuromuscular diagnoses are not uniformly available or were not extracted in this study. Length of stay (LOS) was not modeled as a predictor but may be explored in future analyses. Procedure Class Refined for ICD-10-PCS was used to classify interventions as Minor/Major and Diagnostic/Therapeutic. Major surgery was defined using the Diagnosis Related Group (DRG) assignment. While KID includes indicators for surgical procedures and DRG classifications, variables such as elective versus emergency procedures, prior hospitalization history, hereditary or immunologic conditions, and detailed metabolic or neuromuscular diagnoses are not uniformly available or were not extracted in this study. While age was included as a continuous and categorical variable in regression modeling, subgroup analyses based on developmental categories (e.g., neonates, infants, children) were not performed. Prematurity status and gestational age are also not captured in the KID dataset, limiting analysis of neonatal subpopulations. Major surgery was defined using the Diagnosis Related Group (DRG) assignment (see Table Appendix A.1 for detailed DRG and MDC criteria).

### 2.3. Statistical Analysis

All analyses were conducted using AHRQ’s SAS QI^®^ v2020 software (compatible with SAS 9.4) and associated population files [2]. Logistic regression was used to model the binary outcome of PORF, reporting odds ratios (ORs) and adjusted odds ratios (aORs). Discharges served as the unit of analysis.

Statistical modeling included key hospital- and patient-level covariates. Models assessed associations between PORF and predictor variables, guiding interpretation for quality improvement interventions.

### 2.4. Ethical Review

This study was approved as exempt by the Georgia Southern University Institutional Review Board (Protocol H23359) because it involves de-identified, publicly available secondary data and does not include identifiable human subjects.

## 3. Results

The descriptive analysis of the HCUP KID dataset (2019) provides valuable insights into the distribution and characteristics of hospitals and patients in the sample. Regarding the dependent variable, the PDI in question (PDI 09), it was taken at the numerator level to produce events/occurrences rather than a rate. For reference to inclusion and exclusion criteria regarding PDI09 numerator, publicly accessible specifications can he found on AHRQ’s QI website [9]. In the data, we found that 7.69% of hospitalizations (351,201 cases) had PORF occur, as shown in Table 1. The majority of pediatric discharges were found to be in large hospitals (60.7%) and urban teaching settings (82.3%),

In Table 1, we also analyzed the independent variables according to categories of patient and hospital characteristics that were included in the multivariate analysis. For the continuous variables, we reported the N, Mean and Std. Deviation, whereas for the categorical variables, we have included the N and percentage, as seen below.

### Multivariate Analysis

In the multivariate analysis conducted for this study, our results table (Table 2) showed several significant associations when adjusting for variables. Age, with a mean of 5.76, showed significance (*p* < 0.0001), with an inverse relationship of older patients having a 0.4% decrease in the odds for each additional year of PORF events (AOR = 0.996, 95% CI: 0.995–0.996), signifying a potentially confounded factor played by age in this particular pediatric adverse event. Female gender, on the other hand, did not show significance in the multivariate analysis (*p* = 0.6919).

Regarding racial categories, many of the relationships did not show significance when controlling for other variables, amongst them being patients of Black race or “Other”. Meanwhile, Hispanic, and Native American patients displayed an inverse relationship with 8.2% lower odds (AOR: 0.918, 95% CI: 0.909–0.928) and 5.2% (AOR: 1.052, 95% CI: 1.016 to 1.089) higher odds, respectively, when comparing to White patients and controlling for other variables. Asian/Pacific islander patients were found to have 2.3% higher odds (AOR: 1.023, 95% CI: 1.005 to 1.041) when compared to White patients and controlling for variables.

Among service lines, the multivariate analysis echoed many expectations, with Maternal and Neonatal services as well as Mental Health/Substance use both showcasing lower odds of PORF when controlling for other variables. Maternal and Neonatal had 43.0% lower odds (AOR: 0.570, 95% CI: 0.565 to 0.576), and Mental Health/Substance had 32.9% lower odds (AOR: 0.671, 95% CI: 0.659 to 0.683) when compared to Medical service lines and controlling for other variables. Furthermore, Surgical services demonstrated 51.0% higher odds (AOR: 1.510, 95% CI: 1.478 to 1.544) of PORF events when compared to Medical services and controlling for other variables. Meanwhile, Injury service line was found to not be significantly associated with PORF events when compared to Medical.

In terms of payment source, Medicare and “No charge” remained insignificantly associated with PORF events when comparing to Self-pay and controlling for other variables. However, “Other” payment source was found to be significantly associated (*p* < 0.0001) with PORF with 7.9% higher odds (AOR: 1.079, 95% CI: 1.052 to 1.107) when compared to Self-pay and controlling for other variables. As for Medicaid payments, they had 5.7% lower odds (AOR: 0.943, 95% CI: 0.926 to 0.960), while Private Insurance had 5.5% higher odds (AOR: 1.055, 95% CI: 1.036 to 1.074) when compared to Self-pay and controlling for other variables.

As further expected, major operation on record remained a significant predictor of PORF (*p* < 0.0001), with 17.9% higher odds (AOR: 1.179, 95% CI: 1.157–1.201) when compared to no major operations and controlling for other variables.

With respect to hospital characteristics, small hospitals had 25.1% lower odds (AOR: 0.749, 95% CI: 0.742–0.757), and medium-sized hospitals had 29.2% lower odds (AOR: 0.708, 95% CI: 0.701–0.714) of PORF when compared to large-sized hospitals and controlling for other variables (both *p* < 0.0001). Hospital location and region also showed a significant association with PORF (all *p* < 0.0001), with Rural hospitals showcasing 68.3% lower odds (AOR: 0.317, 95% CI: 0.312 to 0.322) and Urban Nonteaching 60.6% lower odds (AOR: 0.394, 95% CI: 0.389–0.400) when comparing to Urban Teaching hospitals and controlling for other variables. Moreover, hospitals in the Northeast region were associated with 20.5% lower odds (AOR: 0.795, 95% CI: 0.786–0.805), while Midwest hospitals had 17.5% lower odds (AOR: 0.825, 95% CI: 0.816–0.834), and hospitals in the South region showed 13.7% lower odds (AOR: 0.863, 95% CI: 0.854–0.871) of PORF events when compared to the West region and controlling for other variables (all *p* < 0.0001).

Furthermore, hospital ownership was found to be positively associated with PORF, with Public hospitals displaying 14.6% higher odds (AOR: 1.146, 95% CI: 1.128–1.164), and Private non-for-profit hospitals 14.2% higher odds (AOR: 1.142, 95% CI: 1.128–1.156) when comparing to Private investor-owned hospitals and controlling for other variables.

The logistic regression analyses illuminated the multifaceted nature of PORF risk. While some associations showed consistency with expectations when taken into a broadened context, some were a bit telling or seemed to be confounded by other factors, soliciting further investigation.

Race showed significant differences in PORF risk, with different racial categories having varying odds ratios compared to White patients. These associations were also subjected to inverse relationships when considering the simultaneous effects of multiple variables, which suggests a more nuanced understanding of the associations between the variables and merits more in-depth analysis.

It is of note that across analyses, “Medicare” and “No Charge” were both found to be insignificant predictors of PORF, while “Private” insurance seemed to have a positive influence on the odds regardless of variable control. This suggests that certain socioeconomic patient characteristics should be taken into consideration when assessing and mitigating PORF occurrence.

It is also of note, when considering the type of medical complications that entail PORF as an AE, the highly operative nature of the PAE. This was reflected in the analysis, with focus on “Surgical” services as a place of their occurrence, even when other factors are at play.

Furthermore, the type of operation on record was significantly associated with PORF, as major operating room procedures on record were linked to higher odds of PORF across analyses. This suggests that more complex surgical procedures are associated with increased risk and encourages surgeons and healthcare providers to be particularly vigilant when managing high-risk surgeries, and to consider additional preventive measures.

Regarding hospital characteristics, smaller hospitals and hospitals in certain regions were associated with lower odds of PORF. Meanwhile, Public and Private non-profit hospitals consistently showed higher odds of PORF when compared to Private investor-owned hospitals across analyses, suggesting that certain hospital types may be more equipped with PORF prevention measures, further highlighting the important role hospital characteristics play in the occurrence, management, and prevention of PORF.

Overall, the analysis highlights the complex and multifactorial nature of PORF. It demonstrates the importance of considering multiple patient and hospital characteristics when assessing the risk of PORF. The findings can inform healthcare providers and policymakers in identifying quality improvement measures targeting the occurrence of PORF.

## 4. Discussion

**Figure 1:**
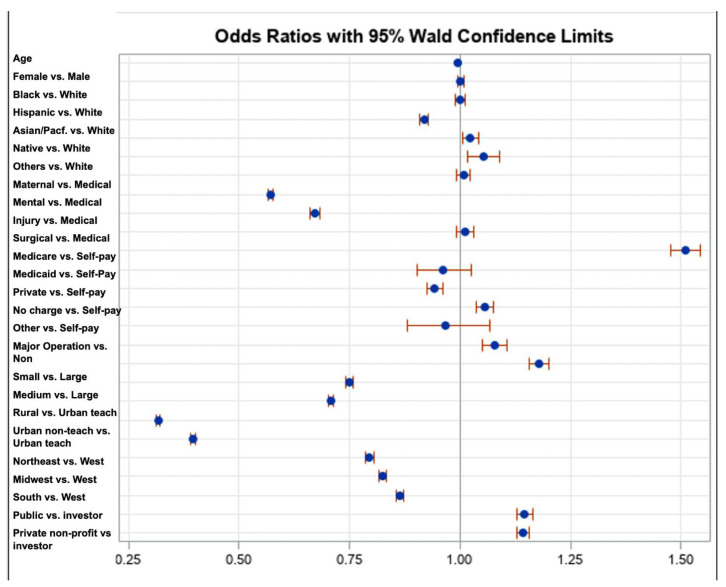
Forest plot: adjusted odds ratios and 95% confidence limits from multivariable logistic regression of pediatric quality indicator—PDI 09. Blue circles represent adjusted odds ratios from the logistic regression model, with red bars indicating 95% confidence intervals.

In this nationally representative analysis of pediatric inpatients, we found PORF to be an infrequent but impactful complication. The overall incidence of PORF in 2019 was 7.96% of pediatric discharges in our dataset, higher than prior estimates of different databases (approximately 1.3–1.4% of NIS datasets in 2012–2014) [4]. Our multivariable model identified several key risk factors that are significantly associated with pediatric PORF, highlighting the multifactorial nature of this adverse event as is reflected in Figure 1. Patient age was significantly associated with PORF risk, with neonates and infants at substantially higher risk than older children. This finding aligns with earlier studies indicating that younger pediatric patients, especially those under one year of age, are prone to post-extubation respiratory failure [7]. Surgical complexity also emerged as a critical factor: patients who underwent major surgical procedures had significantly higher odds of PORF, underscoring the procedure-related risk inherent in this indicator. Notably, discharges categorized in surgical service lines had markedly elevated PORF rates compared to medical admissions, reflecting the fact that PORF is by definition tied to perioperative events. These results reinforce that PORF in children is a multifactorial outcome influenced by patient-specific vulnerability (e.g., age) and the complexity of surgical intervention [1,5,7,8].

Our findings on racial and ethnic disparities in PORF offer a nuanced contrast to prior research. A recent nationwide study by Parikh et al. examined all AHRQ Pediatric Quality Indicators (PDIs) and reported that Black and Hispanic children had higher odds of safety events—particularly sepsis and PORF—compared to White children [3]. In that study, Hispanic ethnicity was associated with increased PORF risk, and children with Medicaid insurance had 45% higher odds of PORF relative to those with private insurance [3]. In our analysis, which focused specifically on PDI 09 (PORF) with a detailed adjustment for patient and hospital variables, we did not observe an increased PORF risk for Hispanic patients. On the contrary, Hispanic children in our cohort had slightly lower adjusted odds of PORF compared to White children, while Native American children experienced higher odds [3]. We found no significant difference in PORF occurrence between Black and White patients. These disparities (or lack thereof) suggest that the influence of race/ethnicity on PORF may be context-dependent. One explanation is that our model included extensive controls for clinical and hospital factors, potentially accounting for some of the variance that in other analyses was attributed to race. Parikh et al.’s approach, which leveraged survey-weighted data for multiple PDIs, may have captured aggregate disparities but did not isolate PORF-specific confounders. The discordance in Hispanic PORF risk between the two studies could stem from differences in inclusion criteria or analytic approach—for example, Parikh’s study relied upon stratified weighting adjustments, whereas our single-year analysis provides a more granular look at 2019 data without weighting. Our use of stratified modeling by hospital characteristics may have further attenuated the apparent racial effects. Importantly, the finding that Hispanic children did not have excess PORF risk in our study should be interpreted cautiously; it may reflect effective management within certain systems or unmeasured protective factors, and it warrants confirmation in other datasets.

Insurance status was another area where our results diverged from prior expectations. We found that children covered by Medicaid had a lower adjusted likelihood of PORF compared to those with private insurance. This is the opposite of Parikh et al.’s report, which found Medicaid patients at significantly higher risk of pediatric safety events including PORF [3]. In our data, privately insured patients and those with “Other” payment sources (e.g., self-pay or government programs other than Medicaid) experienced higher odds of PORF. One possible explanation is that Medicaid-covered children in 2019 may have had greater access to preventive programs or specialized pediatric centers (due in part to Medicaid expansions under the Affordable Care Act) that mitigated their risk [11]. In contrast, privately insured patients might include a subset undergoing very complex elective surgeries (often covered by commercial insurance) at tertiary centers, thus encountering higher inherent risk. Another contributing factor could be differences in data handling; our analysis incorporated hospital-level controls (such as teaching status and region) that may moderate the pure payer effect, whereas Parikh et al. primarily highlighted unadjusted disparities or those adjusted for a more limited set of covariates [3]. It is also possible that many Medicaid-covered admissions were considered newborns, who generally had lower PORF incidence or were classed under the maternal/neonate service line. Furthermore, the role of survey weighting should also be considered; Parikh’s use of weighted national estimates might amplify differences aligned with broader population patterns, while our unweighted analysis treats each discharge equally and may reflect the within-hospital risk after adjustment. Ultimately, these contrasting findings suggest that socioeconomic risk factors for PORF are complex, and that future researchers should be attuned to the importance of building methodological frameworks that are able to capture their data elements.

Beyond patient demographics, institutional factors were significant determinants of PORF outcomes. We observed that large, urban teaching hospitals had higher rates of PORF compared to smaller or rural hospitals. In our data, being in a small community hospital was associated with lower odds of PORF. This pattern likely reflects case mix and referral patterns; high-acuity pediatric cases (e.g., complex congenital surgeries, neurosurgery) tend to concentrate in large urban academic centers, inherently raising the risk profile for those hospitals. Smaller or rural hospitals may perform fewer high-risk surgeries or transfer such patients out, resulting in lower observed PORF incidence. Interestingly, we found regional differences as well; hospitals in the Northeast, Midwest, and South showed significantly lower adjusted odds of PORF compared to those in the West. The West may include a disproportionate share of large children’s hospitals or specific practice patterns that increase detected PORF rates. With respect to hospital ownership, our analysis indicated that public and private non-profit hospitals had slightly higher PORF odds than investor-owned hospitals. This result must be interpreted in context; very few pediatric hospitals are investor-owned, and those that are tend to be smaller specialty hospitals. The higher apparent risk in public and non-profit institutions likely reflects the concentration of complex care cases at major centers rather than ownership effects alone.

Comparing these results to our previous analysis of NBSIs in the same dataset reveals that different pediatric adverse events have distinct risk profiles [9]. For PORF, surgical admission greatly increased risk, while in the NBSI study, surgical patients had reduced infection odds [9]. Additionally, while female sex carried a slight risk increase for NBSI, sex was not a significant factor in PORF. These contrasts illustrate that even within pediatric inpatient safety metrics, one size does not fit all; interventions must be tailored to the specific complication.

Our study contributes evidence that can inform quality improvement (QI) initiatives in pediatric perioperative care. The clear identification of high-risk subgroups—such as infants undergoing major surgeries at large academic centers—allows hospitals to target preventive strategies where they are needed most. For example, enhanced respiratory monitoring protocols and readiness for reintubation may be especially warranted in infants and neonates after lengthy operations. Interventions proven in adult populations, like lung-protective ventilation and conservative fluid management, should be adapted and evaluated in pediatric surgical patients [1]. Ensuring these practices are in place at high-risk centers could reduce the incidence of PORF. Likewise, the finding that certain demographic disparities are context-dependent suggests that equitable care processes can mitigate risk. Hospitals serving vulnerable populations (e.g., minorities or publicly insured children) should invest in staff training and care bundles to preempt respiratory failure—for instance, using proactive postoperative respiratory therapy (incentive spirometry, chest physiotherapy) in children with underlying lung or neuromuscular conditions [1]. From a systems perspective, our results underscore the need for robust preoperative risk assessment tools. Integrating factors like age, procedure type, and hospital setting into a predictive score could help clinicians identify which pediatric surgical patients are most likely to need prolonged ventilation or reintubation. Early identification allows for preventative actions—for example, scheduling such cases first in the day when staffing is optimal, or recovering them in higher-level care units.

In conclusion, this study provides a detailed profile of PORF risk factors in U.S. pediatric inpatients. The insights gained—from patient-level predictors to hospital-level influences—can guide targeted QI efforts and inform evidence-based strategies to both reduce PORF in children and guide future research in the field.

## 5. Public Health Implications and Recommendations

The findings of these studies have significant implications for public health and clinical practice. Understanding the specific risk factors associated with pediatric PORF allows healthcare providers to identify high-risk patients and implement preventive measures tailored to individual needs. Recommendations include optimizing intraoperative ventilation, fluid management, and pain control to reduce the incidence of PORF. Additionally, interventions such as chest physiotherapy and noninvasive ventilation can mitigate risk factors of PORF, such as UPIs, and improve outcomes in pediatric surgical patients.

Public health efforts should focus on enhancing surveillance systems to monitor pediatric adverse events systematically. Standardized reporting mechanisms and comprehensive data collection are essential for accurately assessing the burden of adverse events in pediatric healthcare settings. Furthermore, education and training programs for healthcare providers should emphasize the recognition and management of pediatric safety, ensuring timely intervention and improved patient outcomes. Large academic centers, which shoulder a higher burden of PORF, should be priority settings for implementing these preventive strategies and sharing successful protocols.

## 6. Strengths and Limitations

The strength of this study lies in its large sample size and comprehensive analyses of various patient and hospital characteristics. Utilizing national databases allows for robust assessments of pediatric adverse events and their predictors. The reliance on a standardized definition of PORF (AHRQ PDI) further improves reliability of comparison with other studies and analyses performed on specific adverse events. Nonetheless, as mentioned earlier, not all PORF events are considered preventable, which limits the reliance on these outcome-based comparisons. Moreover, these studies are limited by their retrospective nature, which may introduce biases and limitations inherent to secondary data analysis. As stated also, we could not adjust for some known risk factors (e.g., baseline pulmonary conditions, intraoperative details) due to data limitations, which should be taken into consideration when assessing confounders. Additionally, the reliance on administrative data may lead to underreporting or misclassifying adverse events, potentially affecting the accuracy of the findings. Furthermore, the observational design of these studies precludes causal inference, highlighting the need for further prospective research to validate the identified predictors and interventions.

## 7. Conclusions

This study provides a focused analysis of the patient- and hospital-level characteristics associated with PORF in pediatric inpatient care, using a large, nationally representative dataset. The findings highlight key predictors—including age, race/ethnicity, service line, payer type, hospital region, size, hospital ownership, and teaching status—that contribute to PORF risk. By modeling these factors, the study emphasizes that PORF is a multifactorial outcome, influenced by both patient complexity and institutional structure.

This analysis contributes to ongoing efforts to improve pediatric patient safety by identifying institutional and demographic factors associated with PORF. The findings may support more equitable resource allocation across hospital types and inform targeted quality improvement strategies in perioperative care. While this study is grounded in ICD-10 coding, the upcoming transition to ICD-11 presents opportunities for even more refined case definitions and risk adjustment methods in future research.

## Figures and Tables

**Table 1:** Comprehensive overview of PORF (PDI 09) and key hospital/patient characteristics in the KID 2019 Database.

Variable	Attributes	Frequency	Percent
**PORF (PDI 09)**	Absent	4,214,198	92.31%
	Present	351,201	7.69%
**Hospital bed size**	Small	470,770	15.20%
	Medium	742,057	24.00%
	Large	1,876,456	60.70%
**Hospital location**	Rural	189,298	6.10%
	Urban nonteaching	356,963	11.60%
	Urban teaching	2,543,022	82.30%
**Hospital region**	Northeast	529,073	17.10%
	Midwest	696,645	22.60%
	South	1,183,705	38.30%
	West	679,860	22.00%
**Hospital ownership**	Public	365,784	11.80%
	Private, not for profit	2,382,758	77.10%
	Private, investor owned	340,741	11.00%
**Gender**	Female	1,587,394	51.40%
	Male	1,500,745	48.60%
**Race**	White	1,407,652	45.60%
	Black	513,619	16.60%
	Hispanic	607,329	19.70%
	Asian/Pacific Islander	123,698	4.00%
	Native American	26,306	0.90%
	Other	188,042	6.10%
**Service line**	Maternal and Neonatal	1,716,825	55.60%
	Mental health/substance use	209,939	6.80%
	Injury	97,434	3.20%
	Surgical	219,576	7.10%
	Medical	845,509	27.40%
**Payment source**	Medicare	10,554	0.30%
	Medicaid	1,567,452	50.70%
	Private insurance	1,270,547	41.10%
	Self-pay	131,918	4.30%
	No charge	3,843	0.10%
	Other	100,660	3.30%
**Operation on record**	No operation in record	2,718,390	88.00%
	Major operating on record	370,893	12.00%
**Variable**	**Attributes**	**Mean**	**Std Dev**
**Age**	N = 4,571,036	5.76	7.82

Abbreviations: PORF, postoperative respiratory failure; PDI, pediatric quality indicators; Std Dev, standard deviation.

**Table 2:** Multivariable logistic regression of PORF—PDI 09 by patient and hospital characteristics, 2019.

Variable		Estimate	SE	Wald Test	*p*-Value	Adjusted OR	Wald 95% CI for AOR Lower Upper	
**Intercept**		−1.7917	0.0124	20,964.6656	<0.0001	**0.167**	-	-
**AGE**		−0.00418	0.000298	196.0796	<0.0001	**0.996**	0.995	0.996
**SEX**	Female	0.00150	0.00379	0.1571	0.6919	1.002	0.994	1.009
	Male *							
**RACE**	White							
	Black	0.000486	0.00536	0.0082	0.9278	1.000	0.990	1.011
	Hispanic	−0.0850	0.00521	266.0689	<0.0001	**0.918**	0.909	0.928
	Asian/Pacific Islander	0.0226	0.00914	6.1137	0.0134	**1.023**	1.005	1.041
	Native American	0.0508	0.0177	8.1824	0.0042	**1.052**	1.016	1.089
	Others *	0.00803	0.00789	1.0349	0.3090	1.008	0.993	1.024
**Service line**	Maternal and Neonatal	−0.5617	0.00471	14,217.7550	<0.0001	**0.570**	0.565	0.576
	Mental health/substance use	−0.3991	0.00884	2040.6849	<0.0001	**0.671**	0.659	0.683
	Injury	0.0112	0.0102	1.2020	0.2729	1.011	0.991	1.032
	Surgical	0.4124	0.0111	1385.5081	<0.0001	**1.510**	1.478	1.544
	Medical *							
**Payment Source**	Medicare	−0.0385	0.0329	1.3729	0.2413	0.962	0.902	1.026
	Medicaid	−0.0589	0.00927	40.3335	<0.0001	**0.943**	0.926	0.960
	Private insurance	0.0533	0.00937	32.3885	<0.0001	**1.055**	1.036	1.074
	No charge	−0.0325	0.0492	0.4366	0.5088	0.968	0.879	1.066
	Other	0.0759	0.0130	33.9742	<0.0001	**1.079**	1.052	1.107
	Self-pay *							
**Operation on record**	Major operating room procedure on record	0.1643	0.00953	297.2510	<0.0001	**1.179**	1.157	1.201
**Hospital bed size**	Small	−0.2887	0.00501	3321.6974	<0.0001	**0.749**	0.742	0.757
	Medium	−0.3458	0.00470	5408.6080	<0.0001	**0.708**	0.701	0.714
	Large *							
**Hospital location**	Rural	−1.1499	0.00777	21,923.3961	<0.0001	**0.317**	0.312	0.322
	Urban nonteaching	−0.9302	0.00692	18,057.0116	<0.0001	**0.394**	0.389	0.400
	Urban teaching *							
**Hospital region**	Northeast	−0.2290	0.00603	1443.3234	<0.0001	**0.795**	0.786	0.805
	Midwest	−0.1925	0.00570	1138.1736	<0.0001	**0.825**	0.816	0.834
	South	−0.1477	0.00499	875.6528	<0.0001	**0.863**	0.854	0.871
	West *							
**Hospital ownership**	Public	0.1361	0.00784	301.7575	<0.0001	**1.146**	1.128	1.164
	Private, not-profit	0.1328	0.00637	435.0090	<0.0001	**1.142**	1.128	1.156
	Private, investor-owned *							

Abbreviations: PORF, postoperative respiratory failure; PDI, pediatric quality indicators; AOR, adjusted odds ratio; CI, confidence interval. Note: The * indicates the reference category; bolded values of AORs indicate significant differences compared to the reference category at *p* ≤ 0.05.

## Data Availability

The original data presented in the study are openly available in AHRQ’s HCUP KID database at https://hcup-us.ahrq.gov/db/nation/kid/kiddbdocumentation.jsp.(accessed on 29 October 2022).

## Abbreviations

The following abbreviations are used in this manuscript:

AHRQ	Agency for Healthcare Research and Quality
HCUP	Healthcare Cost and Utilization Project
UPI	unplanned endotracheal tube intubation
KID	Kids’ Inpatient Databases
PDI09	Postoperative Respiratory Failure indicator
PDI	Pediatric Quality Indicator
PCLASS_ORPROC	Procedure Classes Refined for ICD-10-PCS
PORF	Post Operative Respiratory Failure
MDCs	Major Diagnostic Categories
DRG	Diagnosis-Related Group
QI	Quality Improvement
ICD	International Classification of Diseases

## Appendix A. Classification of Major Operating Room Procedures (PCLASS_ORPROC)

### Appendix A.1. The Data Element PCLASS_ORPROC Indicates Whether a Major Operating Room Procedure Was Reported on the Discharge Record [10]

The presence of operating room procedures reported on a discharge record was determined using the Procedure Classes Refined for ICD-10-PCS. Each ICD-10-PCS procedure code on the record was classified into one of four procedure classes:

- Minor Diagnostic: Non-operating-room procedures that are diagnostic (e.g., BW2800Z CT scan of head using high osmolar contrast);
- Minor Therapeutic—Non-operating-room procedures that are therapeutic (e.g., 079030Z drainage of head lymph, percutaneous approach);
- Major Diagnostic—All procedures considered valid operating room procedures by the Diagnosis-Related Group (DRG) grouper and that are performed for diagnostic reasons (e.g., 00B00ZX excision of brain, open approach, diagnostic);
- Major Therapeutic—All procedures considered valid operating room procedures by the Diagnosis-Related Group (DRG) grouper and that are performed for therapeutic reasons (e.g., 021008W bypass coronary artery, one artery from aorta with zooplastic tissue, open approach).

If at least one major diagnostic or major therapeutic procedure was included on the record, then PCLASS_ORPROC was set to 1. More information on the Procedure Classes (https://hcup-us.ahrq.gov/toolssoftware/procedureicd10/procedure_icd10.jsp) is available under Tools & Software on the HCUP-US website (accessed on 29 October 2022).

Prior to the data year 2020, the major operating room procedure indicators were stored in the data element I10_ORPROC.

**Variable**	**Description**	**Value**	**Value Description**
PCLASS_ORPROC	Major operating room ICD-10-PCS procedure indicator	0	No major operating room procedure reported on discharge record
		1	Major operating room procedure reported on discharge record

A. All discharges were categorized into five hospitalization types (i.e., service lines) in the following hierarchical order: Maternal/Neonatal, Mental health/Substance abuse, Injury, Surgical, and Medical. The criteria for identifying the hospitalization types varies across data years.

### Appendix A.2. Beginning in Data Year 2019

Beginning in the data year 2019, the five hospitalization types were defined as follows:

Maternal and neonatal discharges were defined using the following MDCs:

- MDC 14 Pregnancy, Childbirth, and Puerperium;
- MDC 15 Newborn and Other Neonates (Perinatal Period).

Mental health/Substance use discharges were defined using the following MDCs:

- MDC 19 Mental Diseases and Disorders;
- MDC 20 Alcohol/Drug Use or Induced Mental Disorders.

Injury discharges were identified using the following screen:

- Clinical Classification Software-Refined categories for the principal ICD-10-CM diagnosis:
  ○ INJ001–INJ027;
  ○ INJ032.

Surgical discharges were identified by a surgical DRG, the same definition used prior to the data year 2019. The DRG grouper first assigns the discharge to an MDC based on the principal diagnosis. For each MDC, there is a list of procedure codes that qualify as operating room procedures. If the discharge involved an operating room procedure, it is assigned to one of the surgical DRGs within the MDC category.

All other discharges that were not assigned to a surgical DRG were identified as a Medical discharge. If the DRG indicated the information on the record was ungroupable (i.e., not identifiable as medical or surgical), then the discharge was assumed to be medical. This rarely occurred (less than 0.1 percent of total discharges).

It should be noted that discharges with a principal diagnosis of injury or poisoning by intentional self-harm are classified under the Medical service line and not the Mental health/Substance use or Injury service lines [10].
